# User perspectives on systematic data collection regarding back pain managed in general practice – a qualitative study

**DOI:** 10.1186/s12891-022-05613-1

**Published:** 2022-07-19

**Authors:** Sarah Morgan, Alice Kongsted, Birgitte Nørgaard

**Affiliations:** 1 Department of Public Health, University of Southern Denmark, J.B. Winsløws Vej 9B, 5000 Odense C, Denmark; 2 Department of Sports Science and Clinical Biomechanics, University of Southern Denmark, Odense, Denmark; 3 Chiropractic Knowledge Hub, Odense, Denmark

**Keywords:** Back pain, User perspectives, General practice, Nationwide cohort

## Abstract

**Background:**

Back pain is a main driver of disability and the most prevalent reason why people in Demark visit a general practitioner (GP). However, little is known about back pain management in primary care. For new strategies to be sustainable and to accommodate the recommendations for evidence-based practice, patients’ perspectives are paramount to complement clinical expertise and research evidence.

This study aimed to identify recommendations for systematic data collection in a nationwide cohort regarding the management of back pain in general practice from the perspectives of GPs and patients.

**Method:**

We applied an adapted exploratory sequential design using focus groups and individual interviews. Seven GPs and ten patients with back pain participated, and four focus groups and seventeen individual interviews were conducted. Data were analyzed using abductive reasoning.

**Results:**

Both GPs and patients with back pain found that 1) recruitment to a cohort should take place through the GPs, 2) the heterogeneity of patients with back pain and their need for individualized treatment and care should be considered, and 3) data from the cohort should feed into a flowchart or guideline to illustrate a generic patient pathway and visually assist both the patient and GP to obtain an overview and, thus, structure the patient pathway.

**Conclusion:**

GPs and patients with back pain both considered the nationwide cohort with the overall aim to investigate back pain management as being extremely relevant in relation to improve t the patient pathway. User perspectives should be explored and integrated into health care interventions.

## Background

Back pain is an unpleasant condition, and although the prognosis of back pain episodes is good, the prospect of curing persistent back pain is poor [1–3]. With a prevalence of 12.9%—stable since 2005—back pain is the most prevalent cause of adults seeking a physician and for work and activity limitations, bed days among the workforce age population, and disability [4]. Alongside the burden of individuals and healthcare systems is economic burden which has increased during recent decades [4]. Moreover, people with back pain tend to become caught in health care in a cycle of different specialists [5, 6]. Due to there being no curative treatment for back pain, self-management is necessary, which is dependent on collaboration with health care professionals [1]. Because back pain is one of the most common reasons for visiting a general practitioner (GP) [7], these professionals play a core role in the lives of patients with back pain. It has, however, been found that the lack of specific information on pain management service from GPs is perceived as a barrier, and a lack of communication and mutual understanding between patients and GPs might also lead to inappropriate patient pathways and patient dissatisfaction [8]. For the GPs, back pain management might be challenged by the fact that in most cases of back pain, a specific spinal pathology cannot be identified [9]. Back pain tends to be entwined with personal and cultural factors and, thus, inseparable from the social and economic context of the affected peoples’ lives [9].

Large scale data collections are, therefore, required to achieve insights into back pain care, understand the drivers of treatment decisions and plan new strategies, and systematic data collection is recommended [9]. For new strategies to be sustainable, it is, however, paramount that end-user perspectives are explored and integrated [10]. Moreover, to fully accommodate the recommendations for evidence-based practice, patients’ perspectives are of paramount importance to complement clinical expertise and research evidence [11].

### The back pain cohort

Our study was carried out to contribute to the development and design of a national general practice cohort for patients with back pain. The overarching aim of the project is to improve health care for people with back pain through the investigation of the content of care, patient- and clinician-level drivers of variation in care and the decision processes in the management of back pain in Danish general practice. Further, the project aims to investigate how management strategies relate to patient outcomes.

### Aim

To increase end-user value and feasibility, the present sub-study was conducted with the purpose of informing the data collection and contributing to co-creation of the final cohort.

Thus, this study aimed to identify recommendations for systematic data collection in a nationwide cohort regarding the management of back pain in general practice from the perspectives of GPs and patients.

## Methods

This study is reported according to the Consolidated Criteria for Reporting Qualitative Research (COREQ) checklist [12].

### Study designs

We applied an adapted exploratory sequential design using focus groups and individual interviews and, again, focus groups to provide context and enhanced understanding of the findings. We did not include quantitative data as conventional in the sequential analysis; we did, however, collect and analyze the data sequentially [13]; thus, the adapted design was reached.

### Setting and participants

Because the back pain cohort is anchored in general practice nationwide, we planned to include patients and GPs from all five regions in Denmark. However, due to the COVID-19 pandemic, we had to approach recruitment pragmatically. Thus, the members of the steering group for the back pain cohort who were GPs acted as gatekeepers in the GP recruitment process, which took place, therefore, as snowball sampling. Regarding patients, contact was established with key people in two local patient associations (focusing on exercises for back pain), who also acted as gatekeepers during patient recruitment. The two key persons were both listed as contact persons for their patient association and both were a board member, too. Possible participants were then contacted by email or phone (by STM), informed about the study and asked for final acceptance of participation.

A total of 7 GPs (from three regions) and 10 patients with back pain (from one region) were recruited. None of the approached participants refused to participate. In the following, the GPs are labelled GP1-GP7 and the patients as P1-P10.

### Data collection

Through a total of four focus groups, two with GPs and two with patients, as well as seventeen individual interviews, one for each participant, the users' perspectives were uncovered. All interviews were based on a generic semi-structured interview guide (Table 1), prepared specifically for the purpose based on the literature and the project description for the back pain cohort.

**Table 1:** Interview guide (generic)

Core questions
● Information and icebreaking ● Description of the patient pathway for patients with back pain ● Description of the complexity and variation in pathways for patients with back pain ● Description of the ideal patient pathway for patients with back pain ● Discussion of challenges regarding diagnosis, treatment and care for patients with back pain ● How do you think a nationwide cohort could contribute to the patient pathway for patients with back pain? ● Which information do you consider essential for a nationwide cohort for patients with back pain, and how should this information be collected? ● Q/A, debriefing and summing up

To facilitate a safe space and minimise power imbalances, the data collection process was carried out separately for GPs and patients, respectively—yet with similar procedures. A female research assistant (STM) trained in both health care (occupational therapist) and qualitative research (MSc) conducted all interviews. During recruitment, the interviewer was presented for all informants in terms of educational background, affiliation and role in the study.

### Focus groups

Data collection took place from September to November 2021 and was initiated and finalized with a focus group (still separately) to facilitate interactions, reflections and discussions across the participants when they reacted and commented on each other’s perspectives, experiences and understandings. Thus, the outcome of focus groups depended on this social interaction [14–16].

Both the initial and final focus groups with the GPs were conducted online to accommodate the participants' busy working day and enable as many informants as possible to participate. The sessions lasted 60–90 min and were all recorded for the analysis. In the initial focus group, five GPs participated, whereas six participated in the final focus group; two GPs participated in both focus groups.

The participating patients with back pain wanted the focus groups conducted onsite, and one of the informants offered his/her house as a location. In the initial focus group, ten patients participated, whereas eight participated in the final focus group; five patients participated in both focus groups.

The initial focus group focused on the topics and questions presented in Table 1.

During the final focus group, core issues and themes found across the initial focus groups and the individual interviews were presented to the informants, and they were asked to reflect on these findings, discuss them and validate or improve them. Both GPs and patients were presented with their own results only.

### Individual interviews

In between the initial and final focus groups, individual interviews with all participants were conducted, comprising a total of 17 interviews. The main purpose of these individual interviews was to uncover in-depth perspectives and reflections on key findings from the initial focus groups in a safe space.

The individual interviews were conducted from September to November 2021; they lasted 30–45 min and were all recorded for the analysis. All individual interviews were conducted via telephone—both due to the COVID-19 pandemic and to flexibility towards the informants’ time schedules.

### Analysis and reporting

Data were analyzed using abductive reasoning, as described by Revsbæk and Tanggard (2015). During such reasoning, which is neither data nor theory driven, but a ‘breakdown-driven’ process, repeated playbacks of the interviews contributes to further understanding when the empirical material was ‘opened up’ to the listening researcher. Thus, our data material consisted only of the audio-recorded interviews (no transcripts) and unstructured handwritten notes from the interviews – the latter to support recall of immediate impressions and nonverbal reactions. The initial step, listening to the interviews, was carried out by STM. Subsequently, two researchers (STM & BN, both with a health professional background) discussed the findings and underlying patterns, and the selection of quotes was discussed. Abductive reasoning is a pragmatic approach that allows the researchers to take note of situations that may generate a breach of understanding followed by a new understanding that occurs. This research approach is particularly suitable for experienced professionals within the field because it allows the incorporation of prior understandings in the analysis [17].

Following the sequential design, data form each interview round (i.e., initial focus groups, individual interviews and final focus groups) and for GPs and patients, respectively were analyzed separately. As described above, all informants provided feedback on the findings in the sequential design.

### Ethics approval and consent to participate

The study was approved by the Research Ethics Committee of the University of Southern Denmark (21/43008), in agreement with both research ethics and General Data Protection Regulation (GDPR) legislation [18], and all methods were performed in accordance with the relevant guidelines and regulations.

Informed consent to participate in the study was obtained from all participants. Before inclusion, all potential participants received written information about the purpose of our study, contact information, anonymity, confidentiality and the responders’ right to withdraw from the study at any time. Before each interview, this information was repeated orally, and an informed consent was signed by all informants, including consent for publication. According to Danish legislation, no further approval was necessary.

## Results

Corroborating the aim of our study, the results are presented under the headings: *Recruitment, data and data collection* and *Contribution to the patient pathway.* We present the results separately for the GPs and patients, respectively.

### General practitioners’ perspectives

Of the participating seven GPs, three identified as male and four as female. They ranged in age from 31 to 75 years and presented up to 35 years of experience in general practice. All stated being experienced with patients with back pain in general practice.

### Recruitment, data and data collection

Regarding the recruitment of GPs to a nationwide cohort, the GPs suggested various strategies, including Facebook, their professional clusters and the medical doctors in specialist training to become a GP. According to the GPs, Facebook would provide a broad and nationwide recruitment strategy—however, only for GPs actively engaged in relevant Facebook groups.

An even more relevant strategy would be through the professional clusters in which GPs organize nationwide in Denmark. Each of these clusters are organized with a board and a chair, and they could assist by distributing information material regarding a nationwide cohort and arranging oral presentations on meetings.

‘So, I guess that many would say that it is totally in line with that you want to contribute, and the clusters are an optimal place for recruitment, and it is easy to find the boards or chairmen; you can just ask for a mailing list… ‘ (GP1)

Regarding the medical doctors in specialist training to become a GP, the informants stated that it should be at the end of their training (phase 3)—that is, when they are experienced and confident about becoming a GP but still have a bit more time left in their schedule compared to the GPs and, thus, could find the time to contribute to the nationwide cohort.

Regardless of which strategy is chosen, it is considered paramount that the information is provided by a GP—‘… a god and conscientious colleague…’ (GP4)—and that what is expected of the participating GPs is unambiguous.

For a nationwide cohort to be beneficial for the GPs, they suggested striving for maximum variation and, thus, data from as many patients with back pain as possible and across various social and economic conditions. Moreover, they suggested data collection from the beginning of a patient’s pathway from diagnosis to treatment and from both short and longer and more complex pathways. Regarding the specific data, they suggested, among others: description of the first contact to health care, number of contacts to health care per year, description of diagnostic procedures and treatment, coherence, collaboration, satisfaction, well-being and quality of life.

The GPs commented that some of the data for a nationwide cohort could be captured from the patient records if informed consent could be obtained from the patients. Because the GPs document during medical examination and treatment anyway, data capturing was preferred as the data collection strategy; thus, the GPs could document as usual instead of contributing additional data to the cohort. They did, however, acknowledge that heterogeneity in documentation could pose a challenge and that a prerequisite for data capturing would be an agreement on the form and content of the medical records for example in terms of a template.

‘…if a template was available for the medical record that roughly contains what you normally discuss with the patients, it would be as easy as if you document conventionally…’ (GP4)

Hence, they underscored that the GPs participating in a nationwide cohort should not be expected to write ‘long prose texts’, and it would be preferable if they could contribute using a pre-printed template or answering a few questions by checking off.

Overall, the GPs agreed that the patients should provide information to the cohort—because they would possess core knowledge regarding living with back pain.

‘(…) because you can have some patients who do not really have that much pain, but where the impact is great, where it's just about their everyday life, the job, the relationship and everything…’ GP6)

Hence, the GPs considered it relevant that the patients respond to appropriate questionnaires regularly. The patients could access the questionnaires either through a webpage or via a personal email. Diaries were also mentioned as a possible data collection method for patients; in diaries, the patients could document, for example, pain, exercises and drug use continuously and more thoroughly than a questionnaire would allow.

### Contribution to the patient pathway

The GPs underscored the heterogeneity of patients with back pain. However, they also recognized some common features that, in their opinion, increased the relevance of the nationwide cohort. They wanted the cohort to contribute with knowledge about the generic patient pathway, both for the patients doing well and those doing less well over time. They mentioned that data could inform the development of generic patient profiles to indicate specific attention points during medical examination and treatment.

‘… I find it relevant as a tool so that we early in the process can identify whether people will follow a conventional pathway with progress, or we can tell from the beginning that it might be a difficult case to handle…’ GP7)

This mentioned tool could, according to the GPs, be a flowchart or algorithm-based guideline for medical examination and treatment.

‘It would be really nice to have such an algorithm-based guideline (…) well, it would be really nice with a flowchart…’ (GP4)

This flowchart or guideline should act as an illustration of the patient pathway and visually assist both the patient and the GP to obtain an overview and, thus, structure the patient pathway. Furthermore, this illustration could serve as an engaging communication tool during consultations and help align expectations and coordinate decisions. It was, however, highly important for the GPs that a guideline or flowchart should guide an individual and tailored pathway and not act as a ‘one-size-fits-all’ kind of truth.

### Patients’ perspectives

A total of ten patients participated; seven identified as female and three as male, and they were aged 48–73 years. Some were still working, others on either early retirement or conventional retirement. They presented their back pain condition as various forms of arthritis, disc herniation and work accidents; all stated that they had had back pain for several years and all trained regularly in specific back training teams as members of a local gymnastics club.

### Recruitment, data and data collection

The patients found that recruitment to a nationwide cohort through their GP was most convenient. They suggested that information material should be accessible in the waiting rooms for the patients to sign up for the cohort, if interested, or the GP could inform and include the patients during the consultation. They mentioned that if their GP asked them for inclusion, it would positively affect their desire to participate.

The patients also suggested recruitment through local clubs for yoga, water gymnastics or (back) gymnastics. They have all met a lot of peers with back pain in these clubs and anticipated that many would be interested in contributing to a cohort. These clubs could be approached through information material distributed in the teams, through the boards or by an oral presentation at a board meeting or a joint event. To reach the younger group of patients with back pain, they suggested including reflexologists, osteopaths, physiotherapists and chiropractors, as well as pain clinics and gyms in the aforementioned information strategy.

The patients underscored that data should capture ‘… the whole person…’ as this quote illustrates:

“The doctor is so busy; I do not remember that I have been asked how I feel about being in pain, what I could do to not be in pain – besides taking painkillers and doing back exercises…” (P4)

The patients recommended that data should include the pathway on a timeline, pain profile, description of contacts to health care, list of diagnosis, diagnostic procedures and treatment, collaboration among health professionals, who coordinates, knowledge dissemination between health professionals, what is good for your back, determinants of compliance (including economy, coping, health literacy, bright or dark mind, support from relatives), what the patient does him/her self to take care of his/her back, ergonomics at home and at work and personal burden of the back pain (including loss of job, well-being and quality of life).

The patients stated that data collection preferably could be via electronically distributed questionnaires. The ‘not-too-long’ questionnaires should include short and precise questions in clear, plain and respectful language without patronizing the respondents.

“It should be written in a plain language – no Latin expressions that ordinary mortals do not understand, and not the same questions again and again.” (P5)

It should be possible to complete the questionnaire within 20 min, and contact information should be provided. Furthermore, adequate time should be provided for answering the questionnaire.

“I just need to think twice, maybe read the question several times before answering” (P6)

The patients also mentioned dairies as a relevant data collection method (e.g., for documenting pain on a VAS scale and exercises on a daily basis). Finally, the patients found that interviews, for example every 3^rd^ month, would be relevant.

### Contribution to the patient pathway

In the contact with their GPs, it was considered paramount for the patients to be seen by a GP who knows them personally and who knows what they have been through regarding their back pain. The want to be taken seriously and to be seen, heard, met and understood.

‘The doctor who knows you personally is the best doctor ever…’ (P2)

Thus, the patients wanted the cohort to contribute to a better flow during diagnosis and treatment because they experienced too many obstacles and waiting time in their pathways.

They wanted the cohort to contribute with an overview and a guideline or catalogue with inspiration and recommendations regarding treatment and care.

‘Doctors can’t know everything, of course they can’t (...), and they are just as different as we are (…),’ but it is important to know because you are uncertain about what will happen next (…) Yes, because you simply feel let down, it's just like you do not mean anything… it is important to feel taken seriously’ (P9)

The patients wanted the guideline to systematize the pathway and visualize for both patients and GPs where the individual patient is on a timeline and provide information about possibilities for the patients to be active themselves.

‘It is always cool if your GP is familiar with the possibilities in the community.’ (P10)

Furthermore, they wanted the guideline to focus on follow-up and collaboration with the GPs.

## Discussion and conclusion

### Discussion

Both GPs and patients with back pain found a nationwide back pain cohort extremely relevant in relation to improving diagnosis and treatment and also found it relevant that their perspectives were integrated into a future solution. Overall, the similarities between the GPs’ and patients’ perspectives were striking.

Both groups found that recruitment to a cohort should take place through the GPs. Our results thus corroborate the perception of the GP as being a gatekeeper and treatment coordinator for patients with chronic diseases – roles that contribute to both safety and compliance in terms of following the treatment due to a long-standing and trusting GP–patient relationship [19–21]. However, other studies highlight that GPs’ high workload should be considered [22–24], particularly when new tasks are added.

Furthermore, both GPs and patients highlighted the heterogeneity of patients with back pain and the need for individualized treatment and care showing the complexity in care for chronically ill patients. The fact that patients want individualized treatment and care—and GPs want to provide this—is far from new; regardless of the diagnosis, patients want to be seen as individuals, and studies have demonstrated improved health outcomes when patients are involved in the management of their own health conditions and when their individual needs are taken into account, including increases in satisfaction [25], quality of life and mental health [26, 27] and compliance [28]. However, studies have also shown the challenges that GPs struggle with in their encounters with chronically ill patients, including discrepancies between the GPs’ and patients’ judgments [24].

Patients also face various challenges during encounters with their GPs. Multiple studies report on GPs’ lack of time to deal effectively with patients’ concerns and their beliefs, and patients experiencing that their GPs are rushed and tend to be preoccupied with their own agendas under time-pressure constraints. Another challenge for the patients occurs when they do not see the same GP every time and, thus, experience a lack of continuity [24].

Finally, both GPs and patients suggested that knowledge from the cohort should feed into a flowchart or guideline to illustrate a generic patient pathway and visually assist both the patients and GPs to develop an overview and, therefore, structure the patient pathway—both for patients doing well and those doing less well over time. Being positive that both GPs and patients miss this overview and want an evidence-based instrument to structure the pathway and facilitate collaboration, this approach might include some challenges. First, research suggests that GPs might experience a number of barriers towards using guidelines or evidence-based recommendations to guide their treatment of patients with back pain [29–32]. Second, the assumption that it is possible to describe patients in terms of ‘those doing well and those doing less well over time’ might turn out to be rather difficult to substantiate, let alone illustrate in few words in a guideline. However, determining similar outcomes have been proven helpful for risk stratification in UK general practice [33], and a number of clinical guidelines exist and have been found to contribute to, for example, reductions in length of stay and hospital costs [34, 35]. Finally, a structured instrument that even describes complex patient pathways in ‘well’ and ‘less well’ might be a mismatch to the informants’ shared focus on heterogeneity and individualized treatment and care; it would be paramount that the guideline is (only) a guiding framework that leaves room for individualization [36].

### Strengths and limitations

Due to the COVID-19 pandemic, it was challenging to recruit informants as widely (nationally) as initially planned. However, we consider the number of informants appropriate (seven GPs and ten patients with back pain), particularly because patterns emerged early in the data collection process. Furthermore, we believe that the sequential design contributed to nuanced and rich data and also to valid and trustworthy results. On the other hand, we are aware that our informants might be less representative due to our sampling strategy. The GPs were found by snowball sampling, potentially meaning that they represent a more positive group towards both the topic (back pain and systematic data collection) and our method (interviews). Furthermore, the patients with back pain were identified through two local patient associations focusing on exercises for back pain, meaning that they might represent a more active and self-determined segment of patients with back pain. We do, however, rely on our data, and we believe that our results contribute valuable knowledge on both GPs’ and patients’ perspectives on systematic data collection regarding the management of back pain in general practice.

## Conclusion

Both GPs and patients with back pain found a nationwide cohort for back pain relevant, and they appreciated that their perspectives were explored. They found that 1) recruitment to a nationwide cohort should take place through the GPs, 2) the heterogeneity of patients with back pain and their need for individualized treatment and care should be taken into consideration, and 3) data from the cohort should feed into a flowchart or guideline to illustrate a generic patient pathway and visually assist both the patient and GP to obtain an overview and, thus, structure the patient pathway.

## Data Availability

The data collected and analyzed during the current study are not publicly available due to Danish GDPR legislation. However, the corresponding author will be happy to answer any questions about these data.
